# HOW NOT TREATING ADHD IN ADULTS CAN GENERATE CLINICAL PICTURES THAT ARE DIFFICULT TO INTERPRET: A CASE REPORT

**DOI:** 10.1192/j.eurpsy.2023.1605

**Published:** 2023-07-19

**Authors:** N. Mancini, G. Menculini, A. Tortorella, L. Zebi, D. Armellini, P. Lorenzetti, M. Salciarini

**Affiliations:** 1 Department of Psychiatry, University of Perugia, Perugia; 2 Department of Mental Health, AUSL Umbria 1, Gubbio, Italy

## Abstract

**Introduction:**

ADHD is a neurodevelopmental disorder that occurs in childhood and can persist in adulthood in a percentage of cases ranging from 15% to 70% (*Cheung, C. H. et al. J. Psychiatr. Res. 2005; 62, 92–100*). In these cases, if not treated, ADHD symptoms can cause severe dysfunction (*Biederman, J., et al. Am. J. Psychiatry 2000; 157(5), 816–818*) often leading to misdiagnosis.

**Objectives:**

The aim of this case report is to describe the clinical picture of a 26-year-old boy with ADHD and the consequences deriving from the missed diagnosis of the disorder during childhood.

**Methods:**

We report a case of undiagnosed and untreated ADHD and the ensuing consequences.

**Results:**

G.V. is a boy who came to our attention complaining about a vague depressive symptomatology. After psychopathological examination we detected mood instability, with the alternance of phases characterized by deep despair and melancholy and phases of agitation with internal tension and generalized anxiety. He reported a tendency to act on an impulsive basis and an occasional abuse of cocaine together with a daily abuse of high doses of Alprazolam. During the past years the boy had been visited by several psychiatrists who made various diagnoses (borderline or avoidant personality disorder, cyclothymic disorder) and prescribed various drugs but none of these were able to stabilize the psychopathological condition. The clinical history revealed the presence of a pervasive picture of inattention and hyperactivity since childhood which had heavily conditioned the patient’s functioning over time. The inattentive pattern has persisted unchanged over the years, while the hyperactive one has improved leaving room for a stable sense of internal tension and generalized anxiety on which mood fluctuations are cyclically inscribed. A diagnosis of ADHD, combined presentation type, was made by using the DIVA-5. The patient was first prescribed lithium, which was subsequently replaced with valproic acid. After mood stabilization and the reduction of anxious symptoms prolonged-release methylphenidate was added to therapy, obtaining resolution of the clinical picture.

**Conclusions:**

Almost all adults with ADHD exhibit a lifelong pattern of frequent mood swings and irritability. Given that many mental health practitioners are unfamiliar with emotional lability in adult ADHD, a bipolar, or cluster B/C personality disorder is more likely to be considered as the cause of the mood swings (*Fayyad, J. et al. BJPsych 2007; 190, 402–409*). An accurate collection of clinical history can guide the diagnosis and help to address adequate treatment.

**Disclosure of Interest:**

None Declared

